# Integrative analysis of transcriptome, proteome, and ubiquitome changes during rose petal abscission

**DOI:** 10.3389/fpls.2022.1041141

**Published:** 2022-10-19

**Authors:** Chuyan Jiang, Tianhua Jiang, Shuning Deng, Chaoli Yuan, Yue Liang, Susu Li, Chao Ma, Yuerong Gao

**Affiliations:** ^1^ Beijing Key Laboratory of Development and Quality Control of Ornamental Crops, Department of Ornamental Horticulture, China Agricultural University, Beijing, China; ^2^ Beijing Key Laboratory for Agricultural Application and New Technique, College of Plant Science and Technology, Beijing University of Agriculture, Beijing, China

**Keywords:** petal abscission, transcriptome, proteome, ubiquitome, rose

## Abstract

Plant organ abscission is regulated by multiple physiological and biochemical processes. However, the transcriptional, translational, and post-translational modifications occurring during organ abscission have not been systematically investigated. In this study, we report transcriptome, proteome, and ubiquitome data for the abscission zone (AZ) of rose petals collected during petal shedding. We quantified 40,506 genes, 6,595 proteins, and 2,720 ubiquitinated proteins in rose petal AZ. Our results showed that during petal abscission, 1,496 genes were upregulated and 2,199 were downregulated; 271 proteins were upregulated and 444 were downregulated; and 139 ubiquitination sites in 100 proteins were upregulated and 55 ubiquitination sites in 48 proteins were downregulated. Extracellular levels of cell component proteins were significantly increased, while levels within protoplasts were significantly decreased. During petal abscission, transcript levels of genes involved in defense response, transport, and metabolism changed significantly. Levels of proteins involved in the starch and sucrose metabolism and phenylpropanoid biosynthesis pathways were significantly altered at both the transcript and protein levels. The transcriptional and translational upregulation of peroxidase (POD), in the phenylpropanoid biosynthesis, pathway may be associated with deposition of lignin, which forms a protective layer during petal abscission. Overall, our data provide a comprehensive assessment of the translational and post-translational changes that occur during rose petal abscission.

## Introduction

Rose (*Rosa* spp.) is one of the most important ornamental crops worldwide. The timing of petal shedding is a major factor in determining the longevity of the rose flower and is thus important for its economic value. Petal abscission, leading to petal shedding, in response to endogenous and exogenous cues is the terminal stage of petal development (Taylor and Whitelaw, 2001; Tucker and Kim, 2015). The cell separation process preceding shedding occurs in a region of functionally specialized cells known as the abscission zone (AZ) (Taylor and Whitelaw, 2001). Previous genetic and mutational studies have identified many molecular components involved in the transcriptional regulation of petal abscission (Estornell et al., 2013; Kim, 2014; Tucker and Kim, 2015; Ma et al., 2021). Global transcriptional regulation of floral organ abscission has been studied in several plant species, including Arabidopsis (Cai and Lashbrook, 2008), tomato (Meir et al., 2006; Meir et al., 2010), and rose (Gao et al., 2016). These studies have highlighted the critical roles of auxin and ethylene pathway genes during abscission (Meir et al., 2006; Gao et al., 2016). However, little is known about the translational and post-translational regulation of this process.

A decrease in protein levels is a hallmark of senescence and can occur through transcriptional downregulation or through post-translational protein degradation, such as proteosome-mediated degradation triggered by the ubiquitination post-translational modification (Shahri and Tahir, 2014). In petunia and rose, proteome and ubiquitome analyses have demonstrated that ubiquitination participates in protein degradation during petal senescence (Guo et al., 2017; Lu et al., 2019). Further investigation of changes in the ubiquitination profile of proteins during petal abscission could thus deepen our understanding of the molecular regulation of abscission.

**Figure 1:**
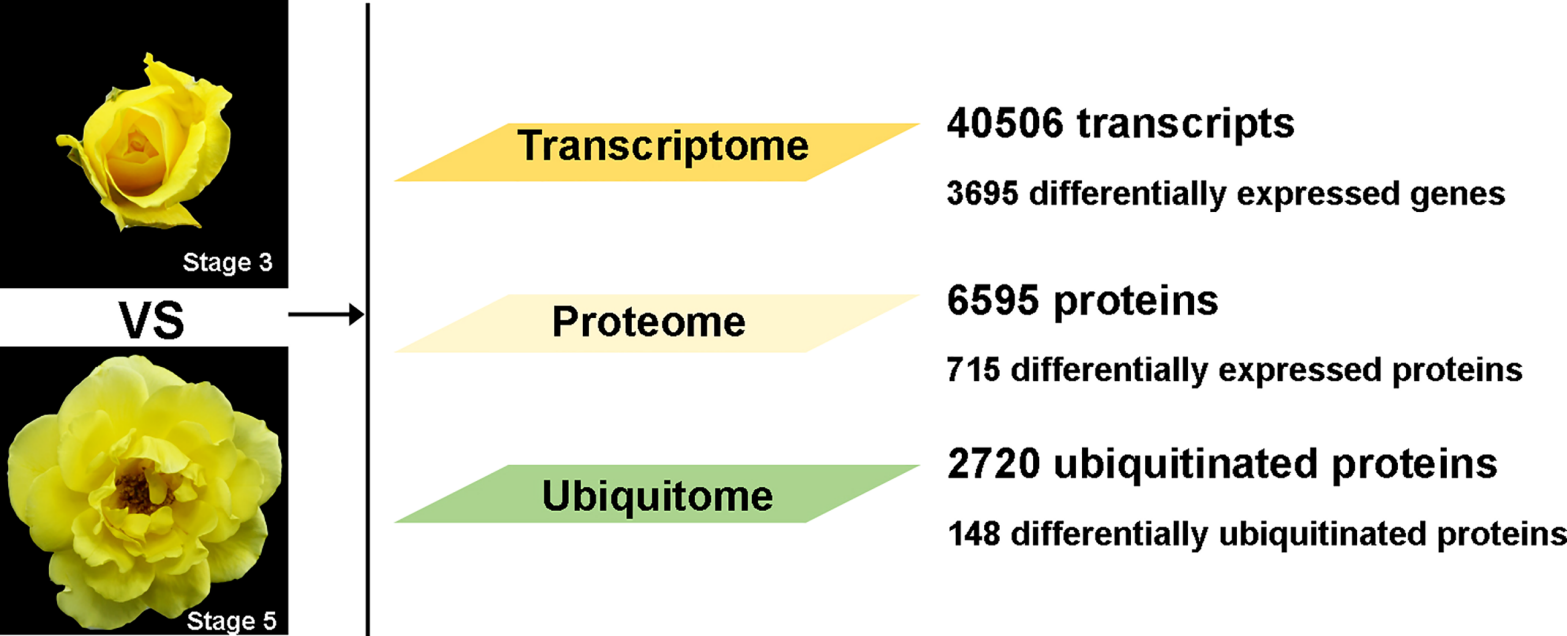
Overall data for transcriptome, proteome, and ubiquitome in petal AZ at flower opening stages 3 and 5.

A previous study has shown that in an abscission-prone rose (*Rosa hybrida* cv Golden Shower), flower opening stages 3 and 5 are marker stages before and after the initiation of abscission, respectively (Liang et al., 2020). In this study, we analyzed petal AZs of ‘Golden Shower’ at stages 3 and 5 for transcriptome changes by RNA-seq, and changes of proteome and ubiquitome using a label-free quantitative strategy involving antibody-based affinity enrichment and high-resolution liquid chromatography-tandem mass spectrometry (LC-MS/MS). In total, we identified 40,506 transcripts including 3,695 differentially expressed genes (DEGs); 6,595 proteins including 715 differentially expressed proteins (DEPs); and 2,720 ubiquitinated proteins including 148 differentially ubiquitinated proteins (**Figure 1**). Our transcriptome, proteome, and ubiquitome data provide a comprehensive analysis of petal abscission at the translational and post-translational levels in rose.

## Results and discussion

### Transcriptome changes in AZ during petal abscission

We obtained transcriptome data sets for stage 3 and stage 5 petal AZ using RNA-seq. In total, we obtained 44.88 GB of clean data and assembled 41,232 transcripts using the Trinity software package. We identified DEGs using a cutoff ratio of >2 or <0.5 (*p*-value <0.05) through a pairwise comparison of expression levels between stage 3 and stage 5 (S5 vs S3) (**Supplementary Table 1**). This yielded 1,496 upregulated and 2,199 downregulated genes (**Supplementary Figure 1A**; **Supplementary Table 1**).

To evaluate the putative functions of DEGs during petal abscission, we determined clusters of orthologous groups of proteins (COG) amongst these genes. DEGs were enriched in pathways related to signal transduction, defense mechanism, and the transport and metabolism of biomolecules such as carbohydrates, lipids, nucleotides, amino acids, and secondary metabolites (**Supplementary Figure 1B**). A previous transcriptome study in the cultivar ‘Gold Medal’ (Gao et al., 2016) had also showed that defense response, transport, and metabolism were enriched during rose petal abscission.

An analysis of DEGs for Kyoto Encyclopedia of Genes and Genomes (KEGG) enrichment showed that the DEGs were enriched in the pathways of starch and sucrose metabolism, plant hormone signal transduction, carbon metabolism, and phenylpropanoid biosynthesis (**Supplementary Figure 1C**). This is consistent with previous transcriptome data for genes involved in petal abscission in the cultivar ‘Gold Medal’ (Gao et al., 2016), which showed that 52 DEGs were enriched in the auxin pathway and 38 DEGs were enriched in the ethylene pathway. Here, we found 46 and 33 DEGs related to auxin and ethylene signaling, respectively (**Supplementary Table 2**), confirming their important roles in rose petal abscission. Previous studies have identified several genes with functions in rose petal abscission whose expression is responsive to petal abscission. The expression profiles of these genes, including *RhERF1* (ethylene response factor1; RchiOBHmChr2g0135921), *RhSUC2* (sucrose carrier2; RchiOBHmChr6g0272341), and *RhARF7* (auxin response factor7; RchiOBHmChr2g0095551), were generally in agreement with the expression profiles that we observed in our RNA-seq date (**Supplementary Table 1**) (Gao et al., 2019; Liang et al., 2020).

### Proteome changes in AZ during petal abscission

Our investigation of proteome changes between stage 3 and 5 petal AZs revealed 715 DEPs with significant changes in abundance, using a threshold of a 1.5-fold difference in abundance (*p*-value <0.05) in stage 5 compared with stage 3. Among these DEPs, 271 proteins were significantly upregulated and 444 proteins were significantly downregulated at stage 5 compared with stage 3 (**Supplementary Table 3**).

**Figure 2:**
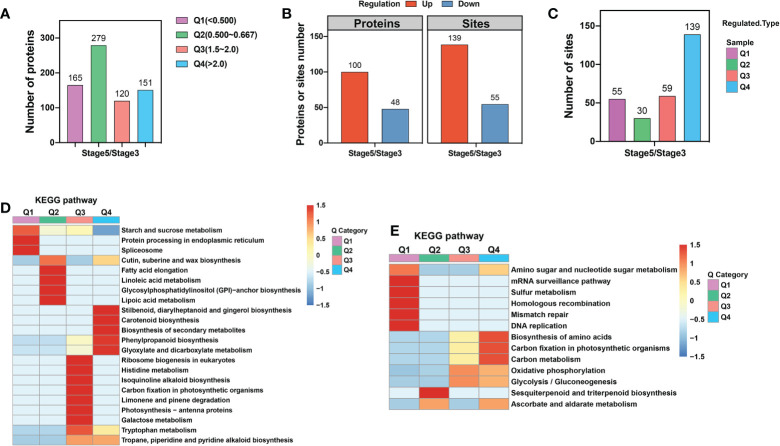
KEGG enrichment analysis of proteome and ubiquitome changes in petal AZ identified through comparison of stages 3 and 5. **(A)** Numbers of differentially expressed proteins (DEPs). **(B)** Numbers of proteins or sites showing significant changes in ubiquitination. **(C)** The four categories of ubiquitination sites defined based on the ratio of stage 5 to stage 3. Q1 represents ratios of <0.667, Q2 represents ratios of 0.667–0.769, Q3 represents ratios of 1.3–1.5, and Q4 represents ratios >1.5. **(D)** KEGG enrichment analysis of proteins with significantly altered abundance. **(E)** KEGG enrichment analysis of sites showing significant changes in ubiquitination.

To elucidate the potential functions of these DEPs, we first divided them into four categories according to their ratio of stage 5 to stage 3 abundance, with Q1 representing ratios <0.5, Q2 representing ratios of 0.5–0.667, Q3 representing ratios of 1.5–2, and Q4 representing ratios >2 (**Figure 2A**; **Supplementary Table 3**). Next, we performed Gene Ontology (GO) enrichment assays for DEPs based on clustering analysis. In the cellular component category, upregulated proteins were enriched in the extracellular component category, including extracellular regions, apoplasts, cell periphery, cell wall, and external encapsulating structure. In contrast, downregulated proteins were enriched in the intracellular component category, including the nucleus, membrane-bounded organelles, endoplasmic reticulum, and endomembrane system (**Supplementary Figure 2A**). These results suggest that during petal abscission, external cellular components of the protoplasm are metabolically active, whereas metabolism within the protoplast slows down. Execution of organ abscission occurs mainly in the middle lamella regions of AZ, and due to the breakdown of the primary cell wall and degradation of pectic polysaccharides (Patterson, 2001; Ogawa et al., 2009; Swain et al., 2011). We also found that a large proportion of upregulated proteins were highly enriched in the metabolic processing of monosaccharides such as hexose, polysaccharides, DNA, and cell wall macromolecules. Downregulated proteins were enriched in the biosynthetic processes of carbohydrates, polysaccharides, membrane lipids, lipoproteins, and glycerolipids (**Supplementary Figure 2B**). These results suggest that cell wall degradation is an important part of petal abscission and that the biosynthesis of organic molecules is reduced during rose petal abscission.

To further delineate the metabolic pathways involved in petal abscission, we mapped the DEPs against the KEGG database (**Figure 2D**). We found that the upregulated proteins were significantly enriched in the metabolism of glyoxylate, dicarboxylate, galactose, and tryptophan, as well as in phenylpropanoid biosynthesis. Downregulated proteins were significantly enriched in starch and sucrose metabolism; protein processing in endoplasmic reticulum, spliceosome; and cutin, suberine and wax biosynthesis (**Figure 2D**).

### Ubiquitination changes in AZ during petal abscission

Next, we examined dynamic protein ubiquitination based on the proteome. The ubiquitome dataset was defined by imputing missing values and normalization of 6,844 ubiquitin-modified peptides and 7,026 lysine ubiquitination (K^ub^) sites in 2,720 proteins, of which 3,718 K^ub^ sites in 1,379 proteins were quantified. Among these quantified K^ub^ sites and proteins, 139 sites in 100 proteins were identified as upregulated ubiquitination sites, and 55 sites in 48 proteins were identified as downregulated ubiquitination sites (**Figure 2B**; **Supplementary Tables 4**). These results suggest that AZ ubiquitination levels are increased during rose petal abscission.

To explore the functional differences between proteins with upregulated and downregulated ubiquitination, we first divided proteins with changed K^ub^ sites into four categories according to their stage 5/stage 3 ratio, with Q1 representing ratios <0.667, Q2 representing ratios of 0.667–0.769, Q3 representing ratios of 1.3–1.5, and Q4 representing ratios >1.5 (**Figure 2C**; **Supplementary Table 4**). GO enrichment analysis showed that, in the cellular component category, proteins with upregulated K^ub^ sites were enriched in proton transport of the vacuolar-type ATPase complex, while proteins with downregulated K^ub^ sites were enriched in membrane proteins (**Supplementary Figure 2C**). In the biological process category, up- and downregulated K^ub^ proteins were enriched in 19 processes. Among them, upregulated K^ub^ proteins were enriched in *S*-adenosylmethionine metabolic and biosynthetic process, ribose phosphate and single-organism carbohydrate catabolic biosynthetic process, and nucleoside and ribose phosphate metabolic process. Downregulated K^ub^ proteins were enriched in hydrogen and monovalent inorganic cation transport and ATP-hydrolysis-coupled transmembrane ion transport (**Supplementary Figure 2D**).

To further delineate the metabolic pathways of proteins with changed K^ub^ sites, we preformed KEGG analysis, and found enrichment in oxidative phosphorylation, nucleotide excision repair, amino sugar and nucleotide sugar metabolism, and glycolysis/gluconeogenesis (**Figure 2E**). Among these, proteins with upregulated K^ub^ sites were enriched in amino acid biosynthesis, carbon metabolism, oxidative phosphorylation, and glycolysis/gluconeogenesis. Proteins with downregulated K^ub^ sites were enriched in amino sugar and nucleotide sugar metabolism, homologous recombination, mismatch repair, DNA replication, and biosynthesis of sesquiterpenoids and triterpenoids (**Figure 2E**).

Next, we investigated the position-specific frequencies of amino acid residues surrounding K^ub^ sites identified in petal AZ using the program Motif-X (**Supplementary Table 4**). Among the 7,026 K^ub^ sites, 5,025 unique sites were assigned to 13 conserved motifs (**Supplementary Figure 3A**), which accounted for approximately 71.5% of the sites identified. Nine of these 13 motifs have been reported previously (Xie et al., 2015; Guo et al., 2017; Lu et al., 2019; Fan et al., 2021), while the other 4 motifs are novel: K^ub^Q, DNK^ub^, DNNK^ub^, and ENNNNNNK^ub^ (where N indicates any amino acid). In addition, we observed that 4 distinct residues were enriched around K^ub^, including alanine (A), glutamate (E), aspartate (D), and glutamine (Q). (**Supplementary Figure 3B**).

### Integrative analysis of AZ transcriptome and proteome

**Figure 3:**
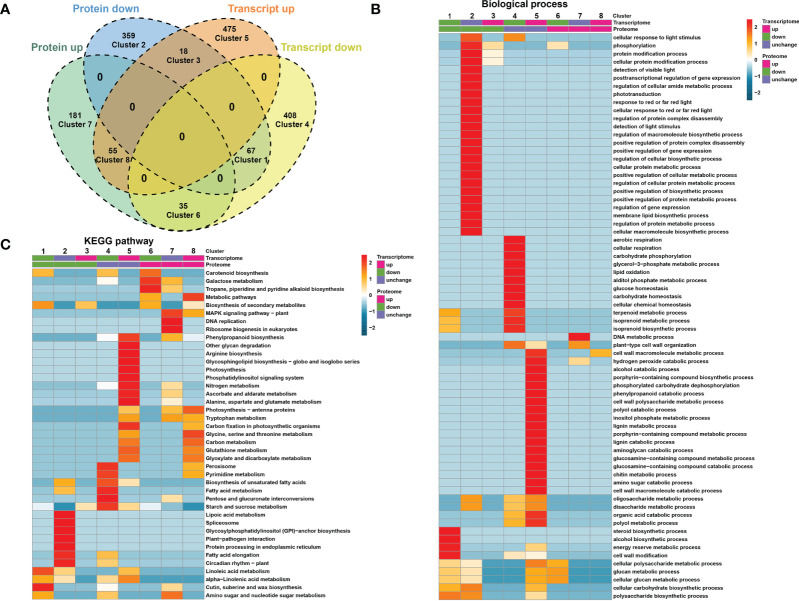
Correlation analysis between DEGs and DEPs. **(A)** Venn diagram showing that DEGs and DEPs are divided into 8 differential expression clusters. **(B)** Gene Ontology (GO)-based enrichment analysis of differential expression clusters in biological process class. **(C)** KEGG enrichment analysis of differential expression clusters.

Comparing the quantitative correlation between transcriptome and proteome data could reveal potential regulatory relationships between proteins and transcripts. Therefore, we performed a correlation analysis between the DEGs and DEPs and divided them into 8 different expression clusters (**Figure 3A**; **Supplementary Table 5**). The results showed that the expression of 67 members was downregulated at both the transcript and protein levels (cluster 1), whereas the expression of 55 members was upregulated at the transcript and protein levels (cluster 8, **Figure 3A**). GO enrichment analysis showed that DEGs/DEPs of cluster 1 were enriched in steroid biosynthetic process, alcohol biosynthetic process, energy reserve metabolic process, and cell wall modification process (**Figure 3B**). KEGG analysis showed that DEGs/DEPs of cluster 8 were enriched in photosynthesis, tryptophan metabolism, and carbon metabolism (**Figure 3C**).

Although not exhibiting changes at the transcript level, at the protein level, 359 and 181 further members were downregulated (cluster 2) and upregulated (cluster 7), respectively (**Figure 3A**). GO enrichment analysis showed that DEGs and DEPs of cluster 2 were enriched in protein modification and metabolic process, macromolecule biosynthetic process, and cellular or protein metabolic processes. DEGs/DEPs of cluster 7 were enriched in DNA metabolic process and cell wall organization (**Figure 3B**). Clusters 4 and 5 contain the largest sets of members (408 and 475, respectively) and exhibited altered expression at the transcriptional level, but not at the protein level (**Figure 3A**).

### Integrative analysis of AZ proteome and ubiquitome

**Figure 4:**
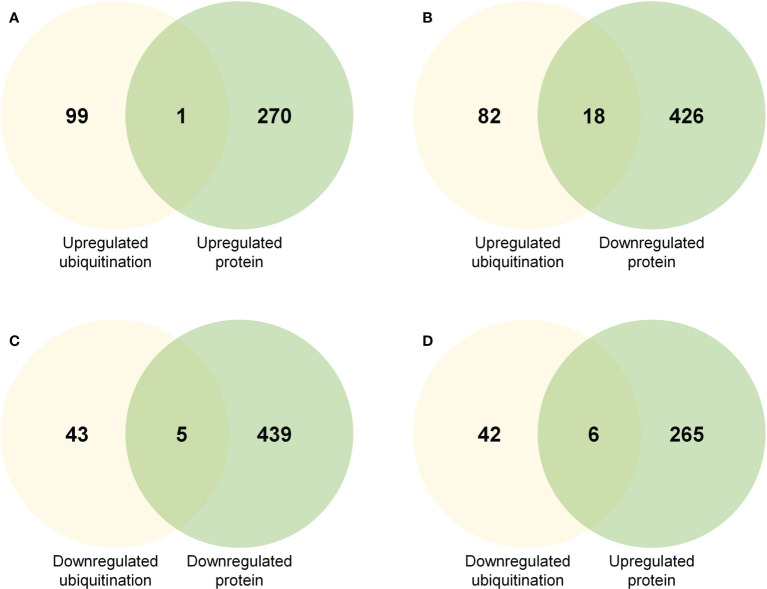
Venn diagrams showing common proteins significantly up- or downregulated in regard to protein abundance and ubiquitination level **(A)** Common proteins in regard to upregulated protein abundance and upregulated ubiquitination level. **(B)** Common proteins in regard to downregulated protein abundance and upregulated ubiquitination level. **(C)** Common proteins in regard to downregulated protein abundance and downregulated ubiquitination level. **(D)** Common proteins in regard to upregulated protein abundance and downregulated ubiquitination level.

To test whether ubiquitination plays an important role in protein abundance and function during rose petal abscission, we compared DEPs with changes in protein ubiquitination (**Figure 4**; **Supplementary Table 6**). We found that the abundance of 18 upregulated and ubiquitinated proteins was decreased, including heat shock protein HSP90, phosphoinositide phospholipase C, and oligopeptide transporter (**Figure 4B**; **Supplementary Table 6**). The abundance of 6 downregulated and ubiquitinated proteins was increased, including pyridoxal 5’-phosphate synthase, ribosome biogenesis factor NIP7, START-like domain, and Bet v I type allergen (**Figure 4D**; **Supplementary Table 6**). Although the technology used in our study may not detect protein abundance or degradation regulated by the 26S proteasome, our results revealed only a few non-regulatory proteins (**Figures 4B, D**), suggesting that the ubiquitination degradation pathway may not play a critical role in rose petal abscission.

### Involvement of phenylpropanoid biosynthesis pathway in petal abscission

**Figure 5:**
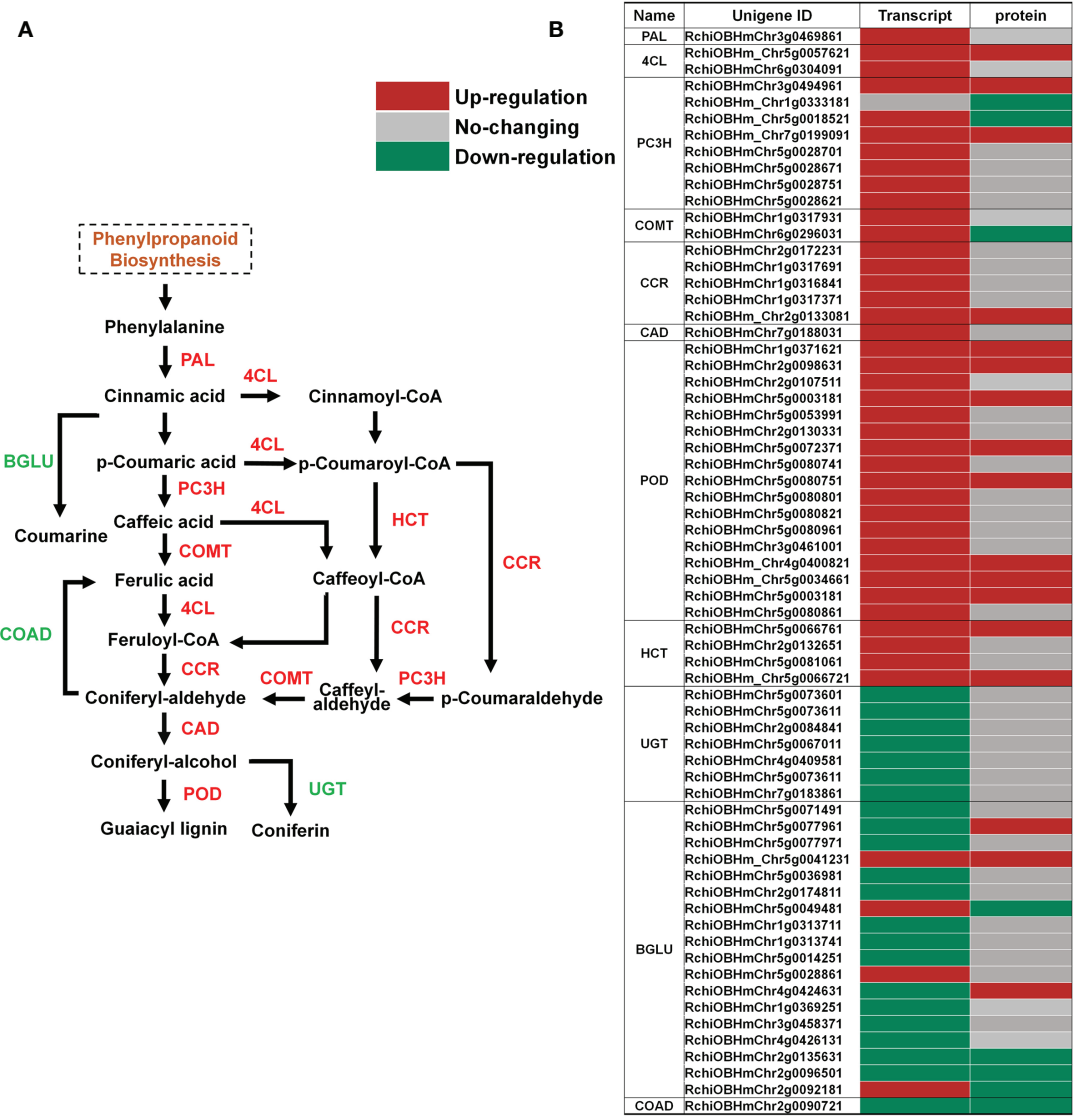
Changes in transcript and protein levels of phenylpropanoid biosynthesis pathway members in AZ during petal abscission. **(A)** Schematic of the phenylpropanoid biosynthesis pathway. **(B)** Expression of genes related to phenylpropanoid biosynthesis at transcript and protein levels.

While cell separation occurs during abscission, the AZ on the mother plant develops a primary protective layer against pests and pathogens, consisting of suberin and lignin deposits (Addicott, 1982). Suberin and lignin are phenylpropanoid-based polymers, and the regulation of the phenylpropanoid biosynthesis pathway has been demonstrated at multiple levels (Vogt, 2010). Our KEGG enrichment analyses showed that the phenylpropanoid biosynthesis pathway was enriched in both its transcriptome (**Supplementary Figure 1C**) and proteome (**Figure 2D**). We observed that among the genes related to phenylpropanoid biosynthesis, the abundance of multiple peroxidase (POD) members was increased at the transcript and protein levels (**Figure 5B**; **Supplementary Table 7**). We validated the expression of genes related to phenylpropanoid biosynthesis in RNA-seq data by RT-qPCR. The results of RT-qPCR were generally in agreement with expression profiles obtained by RNA-seq data (**Supplementary Figure 4**). PODs are involved in the polymerization of monolignols (coniferyl-alcohol) into lignin (**Figure 5A**) (Zhao et al., 2013; Liu et al., 2018). Overexpression of sweet potato POD in tobacco has been reported to cause an increase in the content of phenol and lignin, and enhanced stress tolerance (Kim et al., 2008), and ectopic expression of FaPOD in strawberry (*Fragaria* × *ananassa*) fruit significantly affects lignin biosynthesis and fruit firmness (Yeh et al., 2014). However, the specific role of PODs in organ abscission are yet to be determined.

### Abscission-responsive transcription factors

**Figure 6:**
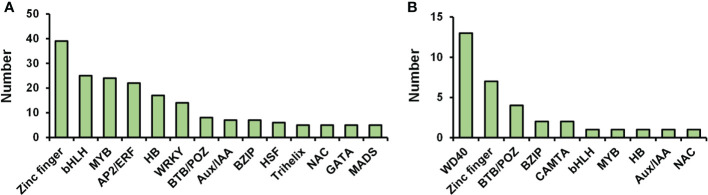
Distribution of transcription factors in transcriptome **(A)** and proteome **(B)**.

Transcription factors (TFs) act critical regulators of transcriptional reprogramming. We identified a large number of TFs in petal abscission transcriptome (208 DEGs) and proteome (33 DEPs). Among these TF related DEGs in transcriptome, the top 10 largest TF families included: zinc finger (39 DEGs), bHLH (25 DEGs), MYB (24 DEGs), AP2/ERF (22 DEGs), homeobox (HB) (17 DEGs), WRKY (14 DEGs), BTB/POZ (8 DEGs), Aux/IAA (7 DEGs), bZIP (7 DEGs), heat stress transcription factor (HSF) (6 DEGs) (**Figure 6A**; **Supplementary Table 8**). This is relatively consistent with previous transcriptome data for DEGs involved in petal abscission in the cultivar ‘Gold Medal’, which also showed same top 10 largest TF families during petal abscission except HSF family (Gao et al., 2016). These results indicated that those TF families may play important roles in petal abscission. Among TF related DEPs in proteome, WD40 (13 DEPs), zinc finger (7 DEPs), BTB/POZ (4 DEPs), bZIP (2 DEPs), calmodulin binding transcription activator (CAMTA) (2 DEPs) are the largest TF families (**Figure 6B**; **Supplementary Table 8**). In addition, we identified 3 TFs related DEPs with changed ubiquitination including 1 WD40 protein, and 2 ARF proteins (**Supplementary Table 8**). These results suggested that petal abscission triggers a complex transcriptional reprogramming.

## Materials and methods

### Plant material

Rose (*Rose hybrida* cv Golden Shower) was used as plant material. The flowers were harvested at opening stage 3 and stage 5 in a greenhouse at the China Agricultural University (Beijing, China). We collected petal AZ samples by excising both sides at the base of petals and receptacles adjacent to the petals as previously described (Liang et al., 2020).

### Total RNA extraction, RT-qPCR, and RNA-seq analysis

Total RNA was extracted using the hot borate method and subjected to RNase-free DNase I (Promega) treatment as previously described (Gao et al., 2016).

For RT-qPCR, first-strand cDNA was synthesized from 1 μg RNA using oligo d(T) and random primers. The RT-qPCR reactions (20 μl) containing 1 μl cDNA were performed by Step One Plus real-time PCR system (Thermo Fisher Scientific). *RhUBI2* was used as the internal control (Liang et al., 2020). The primers used in this study are listed in **Supplementary Table 9**.

For RNA-seq analysis, RNA integrity was analyzed using the Agilent 2100 bioanalyzer (Agilent Technologies). Six RNA samples (three biological replicates for each stage) were sent to Beijing Novogene Bioinformatics Technology Co., Ltd (http://www.novogene.com/) for RNA-seq analysis. The RNA-seq data were processed as described previously (Liu et al., 2022). Clean sequencing reads were aligned using a reference genome sequence (*Rosa chinensis* Old Blush; https://lipm-browsers.toulouse.inra.fr/pub/RchiOBHm-V2/). Genes with at least 2-fold difference and an adjusted *p*-value <0.05 were assigned as DEGs.

### Protein extraction

Petal AZ samples were ground in liquid nitrogen and resuspended in lysis buffer (8 M urea, 1% Triton X-100, 10 mM dithiothreitol, 1% protease inhibitor (Calbiochem), deubiquitinase inhibitors PR-619 (Sigma), and 2 mM EDTA). The mixtures were sonicated and centrifuged at 20,000 *g* (4°C, 10 min) to remove remaining debris. A final concentration of 20% (v/v) trichloroacetic acid (TCA) was added to the supernatants, and the mixtures were kept at 4°C for 2 hours. The mixtures were then centrifuged at 12,000 *g* (4°C, 3 min). The precipitates were washed three times with precooled acetone and dissolved in 8 M urea.

### Trypsin digestion

For digestion, protein solutions were reduced with dithiothreitol (5 mM) for 30 min at 56°C and then alkylated with 11 mM iodoacetamide in darkness for 15 min at 23°C. The solutions were then diluted with 100 mM TEAB to a urea concentration of <2 M. Finally, the protein samples were digested at a 1:50 trypsin/protein ratio overnight or a 1:100 trypsin/protein ratio for 4 hours.

### HPLC fractionation

Tryptic peptides were fractionated by high pH reverse-phase HPLC using an Agilent 300 extend C18 column (5 μm particles, 4.6 mm ID, and 250 mm length) for proteome analysis, and using a Thermo Betasil C18 column (5 μm particles, 10 mm ID, and 250 mm length) for ubiquitome analysis.

### LC-MS/MS analysis

LC-MS/MS analysis was carried out by PTM Biolab Co., Ltd (https://www.ptmbiolabs.com/). Briefly, tryptic peptides were dissolved in 0.1% formic acid, and then loaded onto a reverse-phase analytical column (15 cm length and 75 μm ID). An increasing gradient was applied of 0.1% formic acid in 90% acetonitrile from 6% to 23% over 26 min, 23% to 35% in 8 min, and held at 80% in 3 min, all at a constant flow rate of 400 nL/min on an EASY-nLC 1000 UPLC system (Thermo Fisher Scientific).

Peptides were subjected to an NSI source followed by tandem mass spectrometry (MS/MS) in a Q Exactive™ Plus Quadrupole-Orbitrap™ mass spectrometer (Thermo Fisher Scientific) coupled online to the UPLC. The electrospray voltage applied was 2.0 kV. The *m*/*z* scan range was 350–1,800 for a full scan. Intact peptides were detected in the Orbitrap at a resolution of 70,000. Peptides were selected for MS/MS using a normalized collision energy (NCE) setting of 28, and fragments were detected in the Orbitrap at a resolution of 17,500. A data-dependent procedure that alternated between one MS scan and 20 MS/MS scans was applied with 15.0-s dynamic exclusion. Automatic gain control was set at 5E4.

MS/MS data processing and bioinformatic analysis were performed as previously described (Lu et al., 2019).

## Data availability statement

The datasets presented in this study can be found in online repositories. The names of the repository/repositories and accession number(s) can be found in the article/**Supplementary Material**. RNA-seq data can be found in GenBank under accession number: PRJNA876907. Proteome and ubiquitome data can be found in ProteomeXchange (http://www.proteomexchange.org/) under accession numbers: PXD036672 (proteome) and PXD036665 (ubiquitome).

## Author contributions

CJ and YG conceived and designed the experiments. CJ and TJ performed most of the experiments, SD and SL contributed to the transcriptome assay, CY and YL contributed to the proteome assay. CM provided technical support and conceptual advice. CJ, TJ, and YG analyzed the data and wrote the article. All authors contributed to the article and approved the submitted version.

## Funding

This study was supported by the National Natural Science Foundation of China (32102423), The Construction of Beijing Science and Technology Innovation and Service Capacity in Top Subjects (CEFF-PXM2019_014207_000032), and The improvement plan of scientific research and innovation ability for young teachers of Beijing university agriculture (QJKC2022015), and Science and Technology General Project of Beijing Municipal Education Commission (KM202110020008).

## Conflict of interest

The authors declare that the research was conducted in the absence of any commercial or financial relationships that could be construed as a potential conflict of interest.

## Publisher’s note

All claims expressed in this article are solely those of the authors and do not necessarily represent those of their affiliated organizations, or those of the publisher, the editors and the reviewers. Any product that may be evaluated in this article, or claim that may be made by its manufacturer, is not guaranteed or endorsed by the publisher.

## Supplementary material

Supplementary Table 4 Changes in ubiquitinated proteins and ubiquitination sites.

Supplementary Table 5 Correlation between transcriptome and proteome.

Supplementary Table 6 Correlation between proteome and ubiquitome.

Supplementary Table 7 DEGs and DEPs related to the phenylpropanoid biosynthesis pathway.

Supplementary Table 8 Differentially expressed transcription factors in transcriptome, proteome, and ubiquitome.
