# Supplementary material for: Integrative analysis of transcriptome, proteome, and ubiquitome changes during rose petal abscission
Source: Front Plant Sci. 2022 Oct 19;13:1041141. doi: 10.3389/fpls.2022.1041141 (PMC9627506; doi:10.3389/fpls.2022.1041141)
Supplement: Supplementary file 10 [file DataSheet_10.docx]

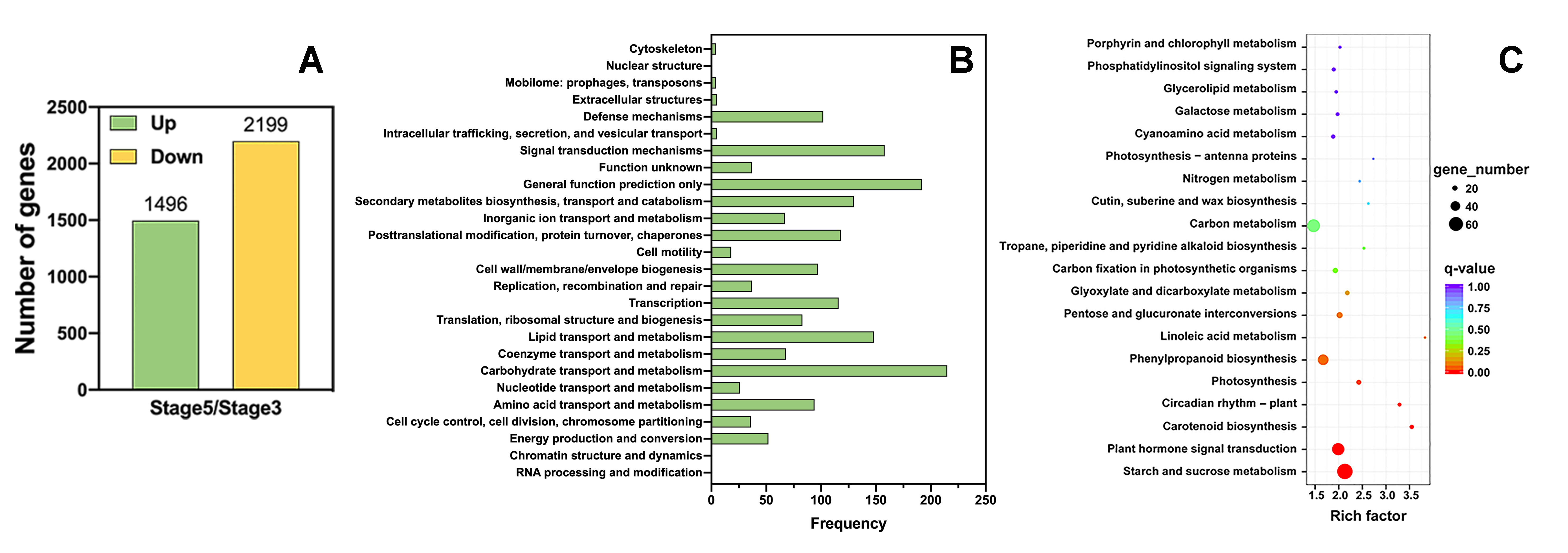


**Supplementary Figure 1. Transcriptome changes in petal AZ identified through comparison of stages 3 and 5.** (A) Numbers of differentially expressed genes (DEGs). (B) Clustering of orthologous groups of proteins (COG) of DEGs. (C) KEGG enrichment of DEGs.

**
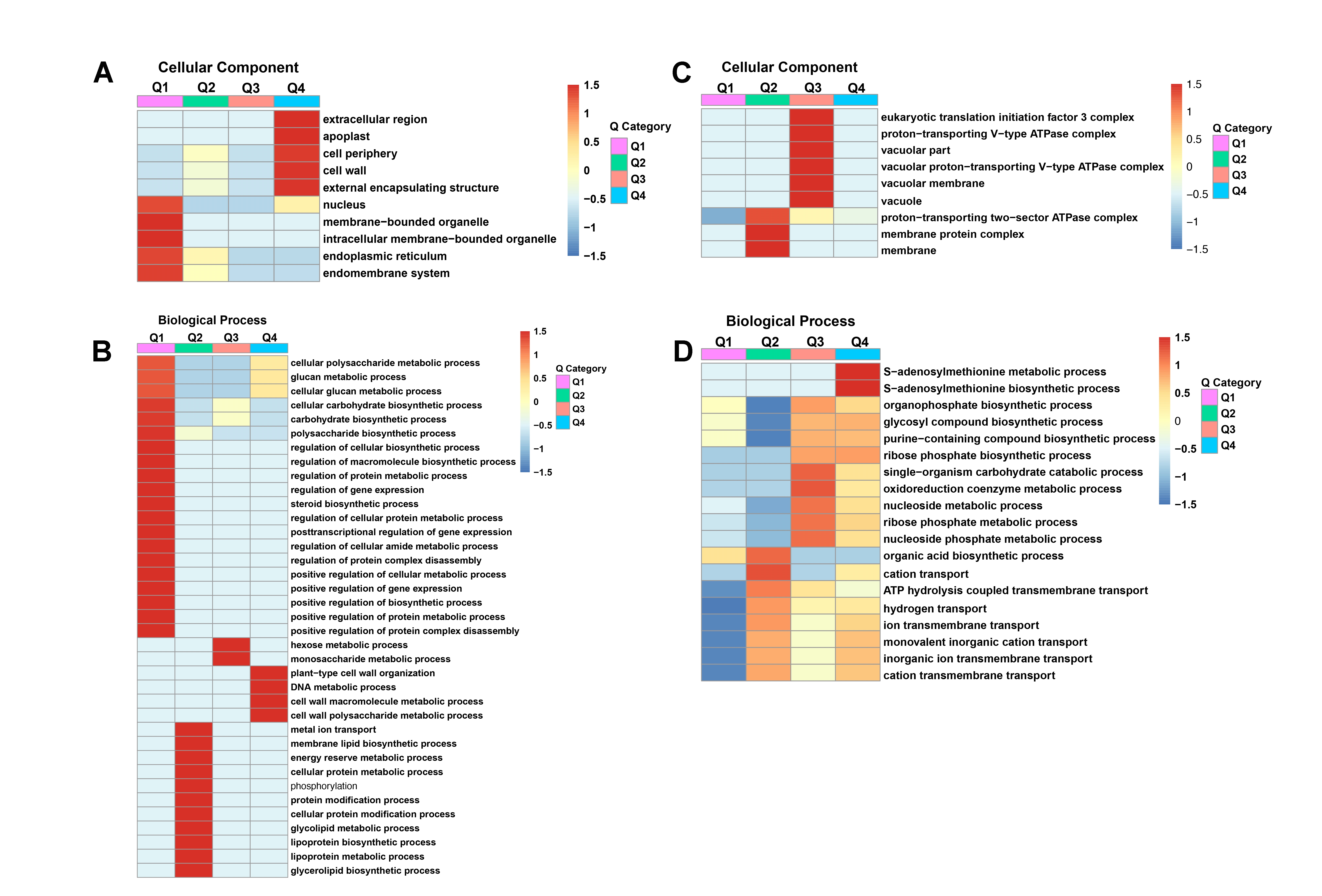
**

**Supplementary Figure 2.** **Gene Ontology (GO)-based enrichment analysis of** **Proteome and Ubiquitome.** (A and B) GO enrichment analysis of proteins showing significantly altered abundance in the cellular component class (A) and biological process class (B). (C and D) GO enrichment analysis of sites with significant changes in ubiquitination in the cellular component class (C) and biological process class (D).


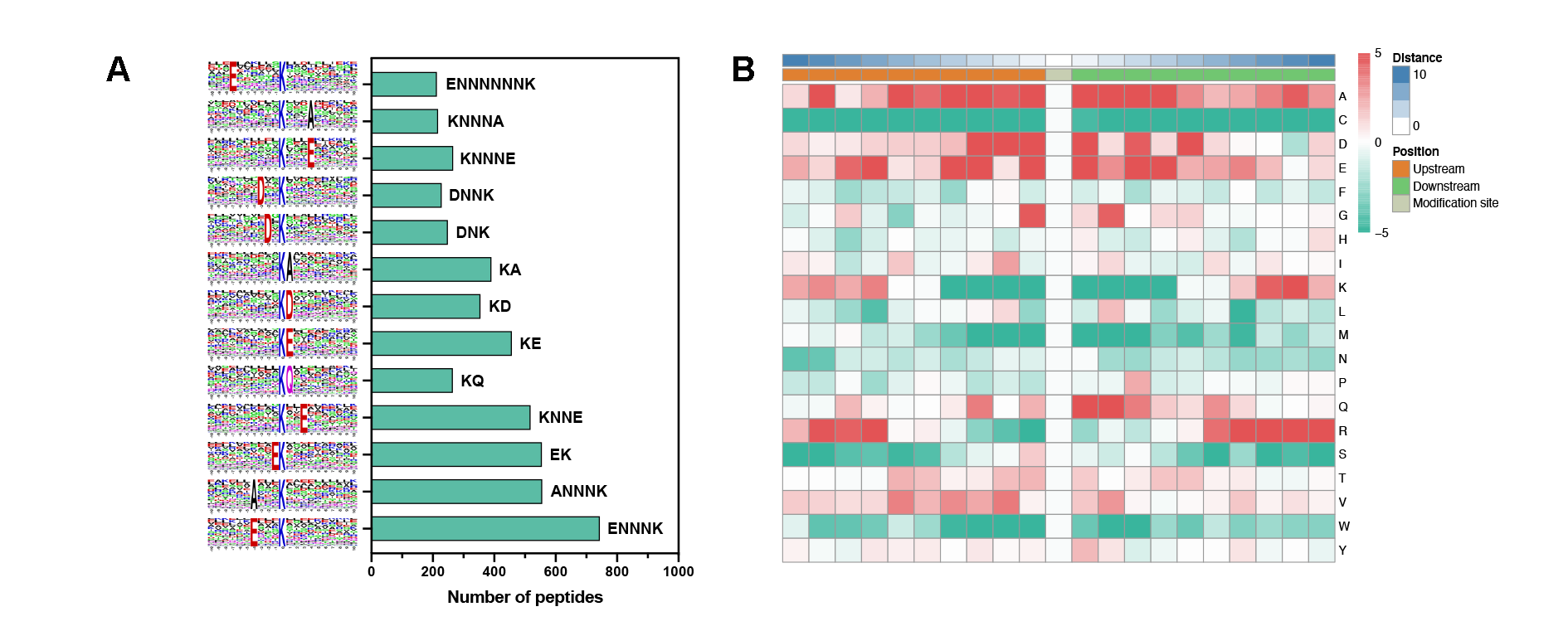


**Supplementary Figure 3. Motif analysis of all K^ub^ sites identified in petal abscission zone.** (A) Number of identified peptides containing ubiquitinated lysine in each motif. (B) Amino acid sequence properties of ubiquitination sites. The heatmap shows significant position-specific amino acids flanking the ubiquitination sites.


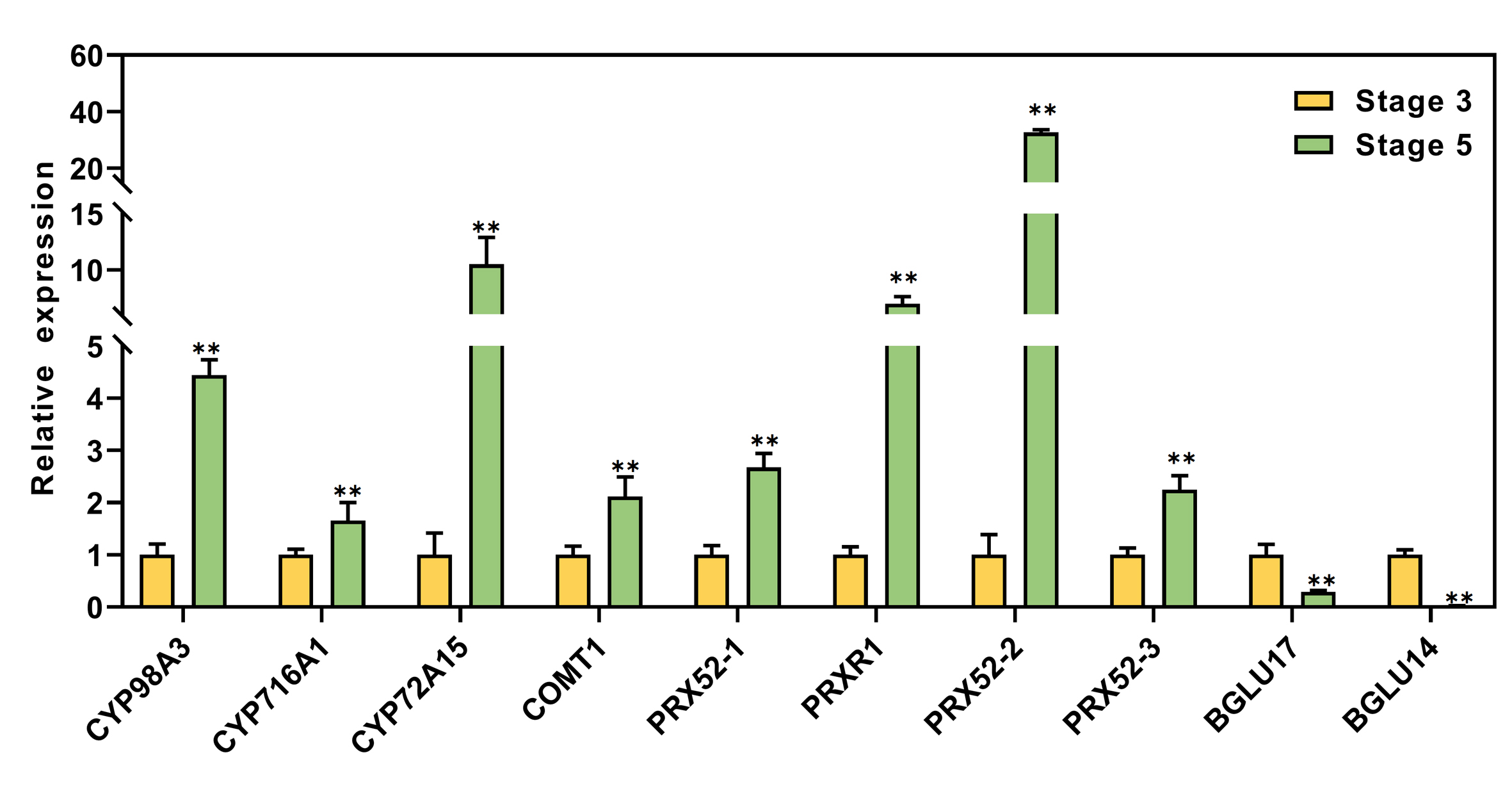


**Supplementary Figure 4. Validation of RNA-seq results by RT-qPCR.** *RhUBI2* was used as the internal control. Asterisks indicate statistically significant differences between stage 3 and stage 5, determined using a two-tailed Student’s *t* test (**P<0.01). *CYP98A3* (RchiOBHmChr3g0494961), *CYP716A1* (RchiOBHmChr5g0018521), *CYP72A15* (RchiOBHmChr5g0028621), *COMT1* (RchiOBHmChr6g0296031), *PRX52-1* (RchiOBHmChr1g0371621), *PRXR1* (RchiOBHmChr5g0003181), *PRX52-2* (RchiOBHmChr5g0072371), *PRX52-3* (RchiOBHmChr3g0461001), *BGLU17* (RchiOBHmChr1g0313741), *BGLU14* (RchiOBHmChr1g0369251).
